# Relationship between *Candida albicans* and *Streptococcus mutans* in early childhood caries, evaluated by quantitative PCR

**DOI:** 10.12688/f1000research.16275.2

**Published:** 2018-12-06

**Authors:** Endang W. Bachtiar, Boy M. Bachtiar

**Affiliations:** 1 Oral Biology and Oral Science Research Center Faculty of Dentistry, Universitas Indonesia, Jakarta, 10430, Indonesia

**Keywords:** Early childhood caries, C.albicans/ S. mutans, Saliva, Dental plaque, qPCR, Indonesian

## Abstract

**Background:** The aim of this study was to analyze the synergistic relationship between *Candida albicans* and *Streptococcus mutans* in children with early childhood caries (ECC) experience.

**Methods:** Dental plaque and unstimulated saliva samples were taken from 30 subjects aged 3-5 years old, half with (n=15, dmft > 4) and half without (n=15) ECC. The abundance of *C. albicans* and *S. mutans* and relative to total bacteria load were quantify by real-time PCR (qPCR). This method was also employed to investigate the mRNA expression of glycosyltransferase ( *gtfB*) gene in dental plaque. Student’s t-test and Pearson’s correlation were used to perform statistical analysis.

**Results:** Within the ECC group, the quantity of both microorganisms were higher in the saliva than in dental plaque. The ratio of *C. albicans* to total bacteria was higher in saliva than in plaque samples (p < 0.05). We observed the opposite for *S. mutans* (p < 0.05). The different value of *C. albicans* and *S. mutans* in saliva was positively correlated, and negatively correlated in dental plaque. Transcription level of *S. mutans gtfB* showed a positive correlation with *C. albicans* concentration in dental plaque.

**Conclusion:** *C. albicans* has a positive correlation with cariogenic traits of *S. mutans* in ECC-related biofilm of young children.

## Introduction

Early childhood caries (ECC) remain the most common childhood oral health problem, globally ^1^, and *Streptococcus mutans* has been known for its important role in ECC development ^2, 3^. However, in recent years, *Candida albicans* has frequently been linked with its synergistic relationship with *S. mutans* in dental plaque recovered from children with ECC ^4, 5^. Consequently, many studies using different methods have been conducted to identify, quantify, and explored the relationship of this fungus with *S. mutans* ^6– 8^. However, a controversial report does exist, where *C. albicans* tends to decrease the cariogenic traits of *S. mutans* in *in vitro* dual-species biofilm ^9^. Therefore, the main purpose of this study was to validate the synergistic relationships between *C. albicans* and *S. mutans*, when growing in caries-related biofilm. For this reason, we requited preschool children with ECC experience, and we used qPCR since it is practice and reliable as a quantitative molecular tool of clinical oral samples ^10^. The fungus and bacterium concentrations in saliva sample were used as control, and we compared the outcomes with those subjects noted as children with free caries (FC).

## Methods

### Subjects

Oral samples were collected from 30 requited preschool children (male and female, 3–5 years old), in two different location located near (about 30 km) to Jakarta, the capital city of Indonesia. The diagnostic of ECC referred to the criteria provided by the American Academy of Pediatric Dentistry, as previously reported ^11^. Two weeks prior to collect the clinical samples, the examiners were calibrated and trained by providing with manual describing study protocol and guidance regarding examination of preschool children. Therefore, only those trained-examiners evaluated the preschool children. The preschool children were recruited to get 15 subjects for each group. Thus, in this study, children were categorized into two different groups; children without any history of caries, including white-spot lesions, (caries free; CF group) and ECC group with decay-missing-filled teeth (dmft) index >4. To be included as subjects in this study, the children were required to be free of symptomatic oral candidiasis, have the absence of any medication therapy during the one month before this study, and have not worn any intraoral appliances. Before oral samples collection, written permission (informed consent) for children to participate was obtained from parents or guardians, according to the guidance provided by the Ethics Committee of Faculty of Dentistry, Universitas Indonesia.

Samples from supragingival plaque, obtained from the selected teeth deciduous (either molar or incisive) were isolated with sterile cotton rolls and pure cotton buds. Samples from the ECC group was obtained by gathering carious biofilm around the affected enamel ^12^, including dentin, as the fungus does not invade carious human dentin ^13^. For the FC group, samples were obtained from enamel in clinically sound gingival areas. In each group, samples collected from molar or incisor, upper or lower teeth were not separated. Therefore, the obtained plaques were pooled to give a single sample for each subject and put in a microcentrifuge tube containing 1 ml PBS (pH 7.4). Unstimulated saliva was collected from all subjects on the same occasion and immediately after the plaque collection, by spitting the saliva into sterile Falcon plastic tubes. The minimum volume of collected saliva was 0.5 ml.

Saliva and plaque samples were immediately cold-transported to the laboratory. For saliva samples, after centrifugation, the sediments were washed three times with 0.5 ml sterile milli Q water between each centrifugation and kept in -80°C until use. Similarly, plaque samples from caries-free or those with ECC were cold-transported to the laboratory and processed as mentioned above, then stored at -80°C until use.

### Quantification of *C. albicans*, *S. mutans*, and total bacteria by qPCR

Bacterial/fungal DNA was obtained by centrifugation each sample in the microtube, using Trizol reagent (Sigma-Aldrich, Dorset, UK). Extracted DNA sample was kept at -20°C after determining the concentration and the quality by Qubit assay reagents (Invitrogen, Carlsbad, CA). The genomic DNA samples were dissolved in Tris-EDTA (TE) buffer and kept in -20°C freeze until used. Further, the DNA samples were quantified through a qPCR reaction with universal primers for the 16S rRNA genes and *C. albicans*/ *S. mutans*-specific primers as shown in Table 1. For PCR-quantification, each sample was run in triplicate on an ABI StepOnePlus Real-Time PCR System with SYBR Green PCR Master Mix (Applied Biosystems, Foster City, CA, USA) according to the manufacturer’s protocol.

**Table 1.** Primers used in this study.

Primer name	Sequences	References
*C. albicans*	Forward:5’-CACGACGGAGTTTCACAAGA -3’	14
	Reverse: 5’-CGATGGAAGTTTGAGGCAAT-3’	
*S. mutans*	Forward: 5’-CCTACGGGAGGCAGCAGTAG-3’	14
	Reverse: 5’-CAACAGAGCTTTACGATCCGAAA-3’	
Universal bacterial 16S rDNA	Forward:5’-GTGSTGCAYGGYTGTCGTCA-3’	15
	Reverse: 5’-ACGTCRTCCMCACCTTCCTC-3’	
*gtfB*	Forward: 5’-AGCAATGCAGCCAATCTACAAAT-3’	14
	Reverse: 5’-ACGAACTTTGCCGTTATTGTCA-3’	

The PCR conditions were run in a final reaction volume of 10 µl, composed of 50 ng of sample DNA and 1 µM of each primer ( Table 1), with thermal cycling condition consisted of a 10 min initial denaturation at 95°C, followed by 40 cycles of 95°C for 15 s and 65°C for 60 s. The cycle threshold (Ct) were determined automatically by the instrument, and a dissociation curve of the amplified fragment set as follow; 95°C for 15 s, 60°C for 60 s, and 95°C for 15 s.

**Figure 1.**
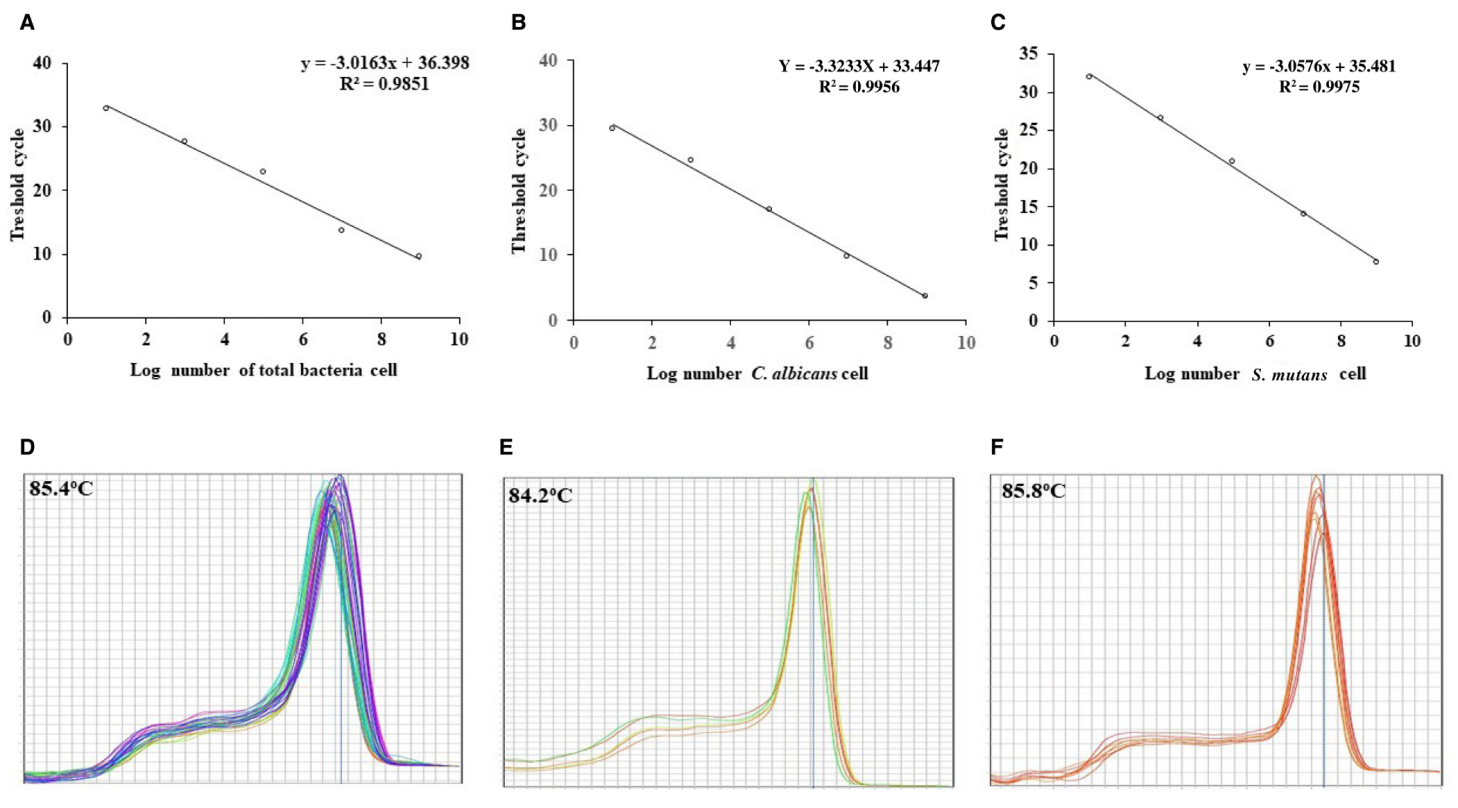
Standard curve construction and melting temperature of qPCR. Standard curves of total bacteria ( **A**), *C. albicans* ( **B**) and *S. mutans* ( **C**). Also shown are melt curve profiles and melting temperatures for total bacteria ( **D**), *C. albicans* ( **E**), and *S. mutans* ( **F**).

Estimating the amount of genomic DNA of both microorganisms tested was determined by constructing standard curves with r ^2^ values for both organisms tested as well as total bacteria ( Figure 1A–C). To do this, we used a 10-fold serial dilution of extracted fungal and bacterial genomic DNA from overnight cultured of *C. albicans* ATCC 10231, *S. mutans* Xc, and *Escherichia coli* JM 107, respectively. The number (CFU/ml) assessed by plating culture dilutions on sabouraud agar, tryptone-yeast extract cysteine with sucrose and bacitracin (TYCSB) agar and Luria Bertani (LB) broth for *C. albicans* ^16^, *S. mutans* ^17^, and *E. coli* ^18^, respectively. The same strains were used as positive control for qPCR. Therefore, quantification of *C. albicans* and *S. mutans* from plaques and saliva achieved by plotting the Ct values against the log of the respective standard curve. In this study, the ratio of *C. albicans* or *S. mutans* in the microbial community, in each sample, was determined as each microorganism proportion to total bacteria. 

For both *C. albicans* and *S. mutans*, the detection limit by qPCR method was determined according to the limit of quantification (LOQ), obtained by the highest dilution of the template of the standard curve. When the Ct value of samples was higher than the LOQ, they would be considered positive, but their melting curve profile should be the same as those of the standards included when running the qPCR.

### qPCR analysis of *S. mutans gtfB* in dental plaque samples

RNA isolation, purification, and reverse transcription of cDNA were performed similarly those in the previous study ^19^. Platinum SYBR Green qPCR SuperMix-UDG (Invitrogen Life Technologies, Carlsbad, CA, USA), passive reference (ROX, Invitrogen), and *S. mutans gtfB* primers ( Table 1), as well as 1 µg of cDNA, were used to quantify the cDNA, and non-transcribed RNA samples were used as control for genomic DNA contamination. The qPCR reaction was run on a similar machine as stated above with cycling conditions consisted of a 10 min initial denaturation at 95°C followed by 40 PCR cycles of 15 s at 95°C, and 60°C for 1 min. The formula of fold change 2 ^-ΔΔCt^ was used to calculate *S. mutans gtfB* gene expression that was normalized to the 16S rRNA, a well-established housekeeping gene ^20^, and *gtfB* expressed in dental plaque of FC group was set to be the control.

### Statistical analysis

The variables for quantification, proportion, and the mean quantitative gene expression were assessed using Student’s t-test, while Pearson’s correlation two-tailed test was used to depict a linear association. Microsoft Excel software was used to perform statistical analysis, and a p-value < 0.05 was considered significant.

## Results

### Quantitative levels of *C. albicans* and *S. mutans* and their proportion in saliva and dental plaque samples

Standard curves were used to determine the corresponding number of microorganism tested while melting peaks were used to assess the specificity of the amplicon using saliva and plaque samples ( Figure 1D–F). 

**Figure 2.**
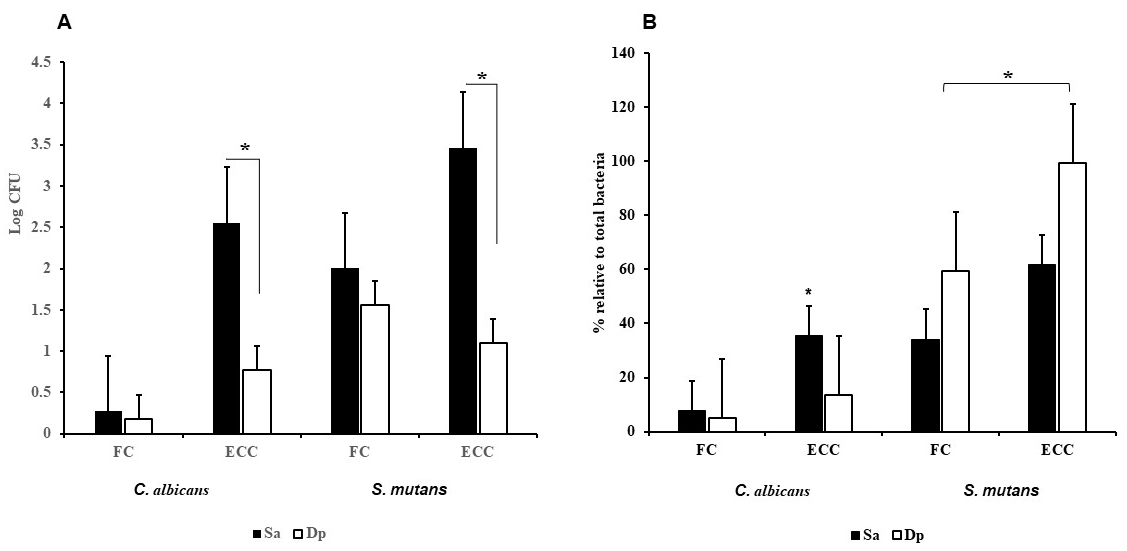
Mean and standard error of absolute numbers ( **A**) and ratios ( **B**) of *C. albicans* and *S. mutans* detected within the same saliva (Sa) and dental plaque (Dp) samples. CF, caries-free; ECC, early childhood caries. *p < 0.05.

In general, this study showed that in all saliva and plaque samples, from either FC or ECC children, both *C. albicans* and *S. mutans* were present. The quantification (log DNA copies) and proportion (% to total bacteria) of *C. albicans* and *S. mutans* in saliva, as well as plaque samples, are presented in Figure 2. Comparatively, it was observed, in either sample tested, a significantly higher number of both microorganisms in ECC children was found more than in those of the FC children (p< 0.05). However, in either group, the quantitative level of *C. albicans*, in the saliva sample was found to be significantly lower than those of *S. mutans* (p< 0.05). When comparing plaque and saliva samples within ECC children, we observed that the load (log DNA copies) of either microorganism in plaque was significantly lower than that in saliva (p< 0.05, Figure 2A). 

Furthermore, we compared the proportion of *C. albicans* and *S. mutans* DNA relative to total bacterial DNA in saliva and dental plaque samples, in FC and ECC children. Within ECC children, there was a significantly higher proportion ratio (Ca/Tb) of *C. albicans* in saliva samples (35.5%), than that in plaque samples (13.5%) (p < 0.05). For FC children the ratio was not statistically different (saliva, 8% and plaque, 5.3%) ( Figure 2B). Similar analysis was carried out for the *S. mutans* proportion ratio (Sm/Tb). The result showed a different trend, with a significantly higher proportion of *S. mutans* in plaque (99%) than that in the saliva (62%) of ECC children ( Figure 2B). The result also showed the proportion of this bacterium to total bacteria in saliva and plaque samples showed a significant difference between ECC and FC children (p < 0.05). Interestingly, there was a trend within dental plaque sample in ECC children, *S. mutans* DNA increased most with increased of *C. albicans*’ DNA ( Figure 2B).

### Association between the value of *C. albicans* and *S. mutans* in dental plaque and saliva of ECC children

**Figure 3.**
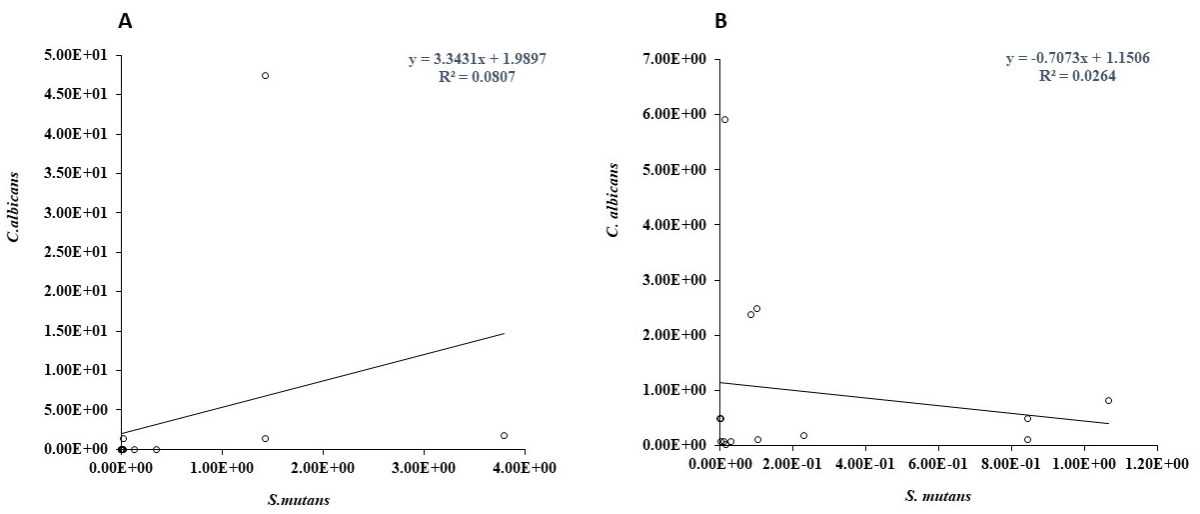
Correlation between *C. albican* and *S. mutans* loads in saliva ( **A**) and in dental plaque ( **B**), in the same subjects. Each circle depicts the value of *C. albicans* (Y-axis) and *S. mutans* (X-axis) in log CFU/ml for each subject.

We further evaluated the possible linear relationship of *C. albicans* and *S. mutans* load or their proportion and ECC experience in the subjects. Pearson correlations coefficient analysis revealed that the association between the numbers of these two microorganisms in saliva was moderate positively significant (r = 0.1, p < 0.05). On the contrary, a weak negative not substantial (r = 0.03, p > 0.05) between the decreasing number of *C. albicans* and the quantity of *S. mutans* in plaque samples observed in the ECC group ( Figure 3A and B).

### Quantification of *gtfB* gene transcription and its correlation with *C. albicans* and *S. mutans* amount in dental plaque

**Figure 4.**
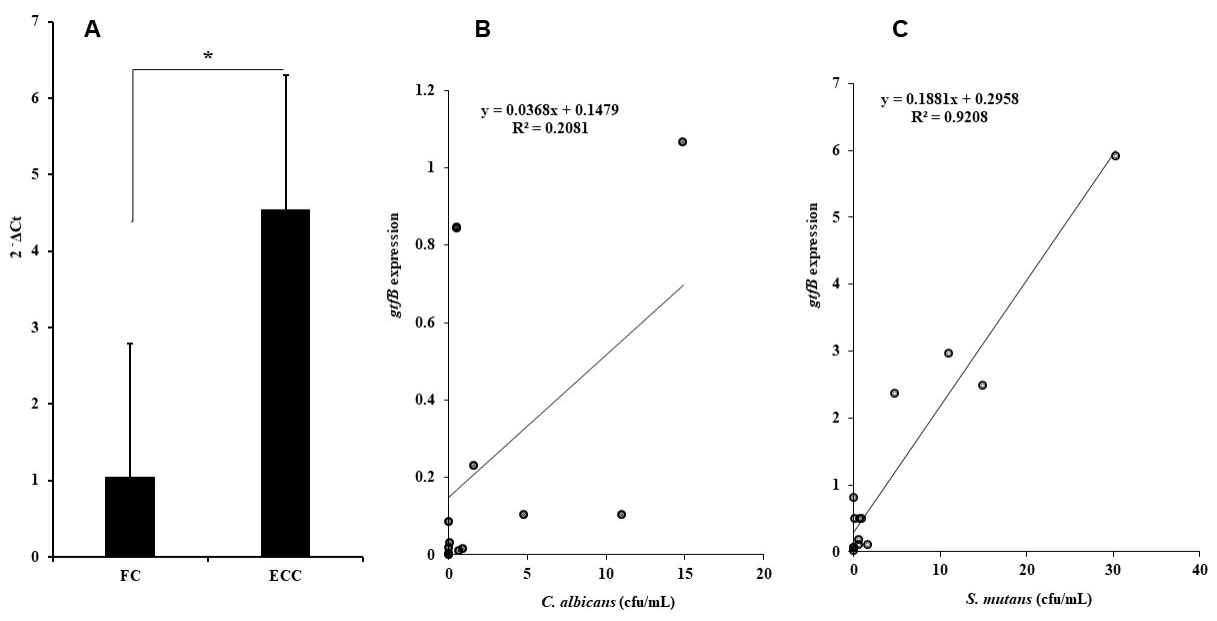
*S. mutans gtfB* gene expression. ( **A**) The fold regulation of *gtfB* cDNA that was normalized to the amount of cDNA of 16S rRNA. ( **B**, **C**) the correlation between *gtfB* transcription rate and the amount of *C. albicans* and *S. mutans*, respectively.

To confirm all the above results, we selected the *gtfB* gene, which has been reported to be mostly involved in the synergistic relationship between *C. albicans* and *S. mutans* in biofilm development ^7^, and compared its expression in each dental plaque of children tested. The qPCR result showed that level of mRNA *gtfB* was induced approximately 4.5-fold in ECC-derived dental plaque samples, and it was a significant difference compared to transcription level of *gtfB* mRNA in dental plaque sampled from children with FC (p< 0.05) ( Figure 4A). Also, *gtfB* transcription levels and the amount of *C. albicans* and *S. mutans* (CFU/ml) in dental plaque of children with ECC showed moderate (r = 0.2) and strong (r = 0.9) positive correlations, respectively, which was statistically significant (p< 0.05, Figure 3B and C).

The raw data associated with this study Data are grouped according to the figure with which they are associated. Click here for additional data file. Copyright: © 2018 Bachtiar EW and Bachtiar BM 2018 Data associated with the article are available under the terms of the Creative Commons Zero "No rights reserved" data waiver (CC0 1.0 Public domain dedication).

## Discussion

Numerous studies ^7, 8, 21^ have supported the idea that ECC may be better understood by focusing on the effect of the functional relationship between species within consortia instead of individual pathogens. This study focused on the relationship between *C. albicans* and *S. mutans*, as these oral microorganisms are frequently detected in the plaque of children with ECC ^12, 22^. Since ECC may indicate a vast proliferation of the cariogenic microorganism, we sought to evaluate the extent to which the amount of *C. albicans* might correlate to the ECC experience. To do this, we used qPCR. This method enabled the quantification of the targeted microorganisms’ genomic DNA from oral samples ^23^. Thus, we examined the similarities and differences by comparing the amount and ratio to total bacteria of each microorganism in saliva and dental plaque samples, and verified their correlation with the occurrence of ECC. In general, we observed that the oral cavities of preschool children in this study, with or without ECC, are colonized by yeast ( *C. albicans*) and bacteria, as represented by *S. mutans*. Overall, the fungus presence was always simultaneously detected with *S. mutans*, although children with ECC had a higher amount of *C. albicans* and *S. mutans* in their oral cavity, compared to those children with FC. As expected, in addition to saliva, these oral microbiotas commonly exist together in an ECC-related biofilm.

Our observation is in accordance with results from other studies, which found that in addition to *S. mutans* as a specific cariogenic bacterium ^24, 25^, *C. albicans* can be part of the dental lesion ^26, 27^. Moreover, previous studies showed strong synergism when *C. albicans* and *S. mutans* co-existed in biofilm, suggesting that this co-existence enhanced their virulence ^7, 8, 28^. However, our data showed, the fungus was detected at lower levels in dental plaque, compared to *S. mutans*. This support the previous *in vitro* study ^29^, which found that the presence of *C. albicans* might favor the extensive colonization of *S. mutans* in dental biofilm.

The causes to generate site specificity bacteria proportion are believed to include local sucrose concentration in the oral cavity ^30, 31^. In addition to sucrose ^32^, many factors may link to the presence of *C. albicans* in children oral cavity. These include infection at birth, baby’s feeding bottles, infected pacifiers, and carious teeth ^12^. We speculate that a high cariogenic diet might influence the interaction between *C. albicans* and *S. mutans* in these children tested. In turns, it becomes critical for ECC, since the presence of sucrose in the children oral cavity may lead to the ability of this species to grow within structured microbial biofilm ^33, 34^. Further studies to obtain data regarding cariogenic diet are therefore necessary.

The presence of yeast and bacteria is one of the local factors that contributes to the etiology of ECC ^7^. To obtain an overall insight into the impact of simultaneous participation of *C. albicans* and *S. mutans* when detecting together in each sample tested, we compared the percent proportion of *C. albicans* or *S. mutans* relative to total bacteria. As expected, the proportion of *C. albicans* was higher in saliva than in carious plaque among children with ECC. Other studies have reported this phenomenon, where *Candida* species were frequently isolated more, qualitatively and quantitatively, from saliva than from dental plaque ^^35^^ and subgingival samples ^36^. During in saliva, this fungus might act as a bridge for oral bacteria to adhere to a mucosal surface, a mechanism that may protect this bacterium from being removed by salivary flow and swallowing ^21^. On the other side, biofilm formation is vital for *C. albicans* to survive as a pathogen, which involves attachment, colonization, and development of structural biofilm integrity composed of yeast and hypha ^37, 38^. Our data illustrate that co-adhesion between *C. albicans* and *S. mutans* in cariogenic biofilm is one mechanism by which the fungus, in yeast form, survives in the oral cavity ^39, 40^. Although the fungus morphology was not observed in this study, it has been reported by other studied that hypha morphology is not crucial for the *C. albicans*– *S. mutans* relationship when they grow in multispecies dental biofilms ^41– 43^. Therefore, in addition to the morphology, both the number and proportion of salivary *C. albicans* influence the fungal–bacterial relationships, which further increases the risk of caries. Additionally, the flushing effect of saliva, as part of innate defense mechanism, might contribute to decrease *Candida* adherence to the oral surface ^44^, including tooth surface. Thus, the quality and quantity of saliva have an essential role in maintaining fungus behavior, as commensal or pathogen ^45^.

We observed that the proportion of *S. mutans* was higher in dental plaque than in saliva, and there was a tendency for the percentage of *C. albicans* to be lower in carious plaque, where the proportion of *S. mutans* increased. This observation indicates that *S. mutans* has an active role in orchestrating the development of cariogenic biofilms ^46, 47^. This species has an essential part in attenuating the virulence of the fungus ^48^ by interfering with the fungus transition, from yeast to hypha form when these oral microflorae interact and grow in biofilm ^49, 50^. This result further supported by the data of correlation analysis, in which the fungus-bacterium concentration in dental plaque sample showed a negative association, although a positive correlation was found in saliva sediment. This suggests that the ECC rate may not be connected to the quantity of *C. albicans* involves.

 One of the mediators for the synergistic relationship between *C. albicans* and *S. mutans* is the streptococcal GtfB enzyme ^7, 32^. Our finding indicates that cariogenic biofilm developed in ECC children accompanied by the increased transcription level of *gtfB* mRNA and enhance of *S. mutans* growth in dental plaque derived from children with ECC. In a clinical situation, this observation is relevant, since GtfB is the enzyme that synthesizes glucan polymers from sucrose ^6^. Thus, result of this study suggests that a high sucrose concentration, which is critical for the development of dental caries, might exist in the children oral cavity. Future clinical studies regarding diet-associated ECC risk factor are thus recommended. Collectively, this study indicates that although the proportion of *C. albicans* was less in ECC-associated biofilm, it may support facilitation of a fungal-bacterial synergistic relationship. Yeast cells could be used by *S. mutans* to promote fitness and the bacterial survival, as shown by enhanced transcription level of *gtfB*, which reflects that more extracellular polysaccharides were produced to promote the fungal-bacterium relationship in caries-related biofilm ^7^.

The results of the present study cannot explain the reason for such an association. However, at least this study provides information on ECC experience among preschool children. The presence of *C. albicans* in dental plaque and saliva could be merely an indicator of oral health conditions and the high carbohydrate intake among the young children selected in this study, which might confer a survival advantage for *C. albicans* and it is favorable for ECC development. More studies regarding the involvement of *gtfB* expression, and how it relates to oral health conditions as well as cariogenic intake in Indonesian preschool children, are needed.

There is some limitation in this study. First, the primary disadvantage of qPCR used in this study is its inability to separate and quantify the viable from nonviable cells. This technique may result in false positives or an overly high estimation, as all DNA extracted from life or dead *C. albicans*, or *S. mutans* cell will be amplified. Since the number of viable cells is especially significant for diagnosing and monitoring disease, adding cell viability information to qPCR-based diagnostics should be considered. Second, the number of children involved in this study was small (15 subjects per group) because of difficulty in sample collection, primarily to obtain the plaque on the dentin surface.

## Conclusions

This study shows that *C. albicans* contributes to increasing concentration of *S. mutans* by inducing the expression of *gtfB* mRNA in ECC-related biofilm. Therefore, results from this study would be useful as a starting point to consider *C. albicans*, as a potential target in prevention programs to reduce the high rates of ECC in individuals or groups of young children. Moreover, since the information regarding oral hygiene habit or diet, provided by the accompanying guardian are difficult to be understood, as found during this study, future studies involving preschool children may wish to involve examiners who are more experience in deal with such parents/guardians when planning studies.

## Data availability

The data referenced by this article are under copyright with the following copyright statement: Copyright: © 2018 Bachtiar EW and Bachtiar BM

Data associated with the article are available under the terms of the Creative Commons Zero "No rights reserved" data waiver (CC0 1.0 Public domain dedication). 

**Dataset 1. The raw data associated with this study.** Data are grouped according to the figure with which they are associated. DOI: https://doi.org/10.5256/f1000research.16275.d221304 ^51^.
