# Supplementary material for: Relationship between Candida albicans and Streptococcus mutans in early childhood caries, evaluated by quantitative PCR
Source: F1000Res. 2018 Dec 6;7:1645. Originally published 2018 Oct 16. [Version 2] doi: 10.12688/f1000research.16275.2 (PMC6221075; doi:10.12688/f1000research.16275.2)
Supplement: Peer review history

# Peer review: Referee response for version 1

**DOI:** 10.5256/f1000research.17775.r39489

**Published:** 2018-11-06

**Authors:** Shahida Mohd-Said

**Affiliations:** 1 Centre for Restorative Dentistry, Faculty of Dentistry, National University of Malaysia, Kuala Lumpur, Malaysia

This paper reports an interesting and relevant study on ECC in children. The methods and contents of the papers are current, relevant and well-structured. However, some improvements could benefit the presentation including checking on grammar and typos, some revisions to sentence construction, highlights on the significance of this study, how the findings can be important to update of knowledge and current management of ECC, and comparison of data from Indonesia with other populations to perhaps illustrate novelty and significance to current available data.

I have read this submission. I believe that I have an appropriate level of expertise to confirm that it is of an acceptable scientific standard.

# Response

**Published:** 2018-11-08

**Authors:** Endang Bachtiar

Dear reviewer,

I am pleased to resubmit for publication the revised version of **Relationship between *Candida albicans* and *Streptococcus mutans* in early childhood caries, evaluated by quantitative PCR.**

We appreciate all the insightful comments provided by the reviewer. Based on the reviewer’s guidance, includes a number of positive changes, we endeavoured to improve the fit of the paper with the journal.

We hope that these revisions improve the paper such that the editor and reviewer now deem it worthy of publication in F1000 Research. Thank you for taking the time to help us improve the paper.

Sincerely,

Authors:  Endang W Bachtiar and Boy M Bachtiar

Responses to reviewer #1:

We have fixed all grammar,  typos, and some revisions to sentence construction. Furthermore all responses to the suggestions have been added in the manuscript in the yellow highlight sentences.

# Peer review: Referee response for version 1

**DOI:** 10.5256/f1000research.17775.r39491

**Published:** 2018-10-31

**Authors:** Zamirah Zainal-Abidin

**Affiliations:** 1 Centre for Craniofacial Diagnostics and Biosciences, Faculty of Dentistry, National University of Malaysia (UKM), Kuala Lumpur, Malaysia

Methods:

1. The authors did not mention how the examiners were calibrated, in order to reduce the inter- and intra-examiner variability when examining the preschool children, before grouping them into the early childhood caries and caries free groups.
2. The authors may have been able to collect more information on the oral hygiene habits or dietary intake of the subjects, before taking the oral samples. 

Results:

1. In the caption for Figure 2, the symbol *p<0.05 should be indicated clearly. For Figure 2A, it is to show that there is a statistically-significant difference between the *C. albicans* in saliva and dental plaque in the ECC group, and a statistically-significant difference between the *S. mutans* in saliva and dental plaque in the ECC group. The use of top square bracket ( ⎴ ) with an asterisk would be better to indicate which parameters are statistically compared.
2. For Figure 4A, the use of top square bracket ( ⎴ ) with an asterisk would be better to indicate the parameters which are statistically compared.

Discussion:

1. In paragraphs 3 and 7, the authors speculated that a high cariogenic/high sucrose concentration diet might influence the interaction between *C. albicans* and *S. mutans* in the tested subjects. The authors may have been able to draw a better suggestion if the information on the oral hygiene habits or diet is obtained during the recruitment of the subjects (See my comment in Methods (2)).
2. In paragraphs 3, 7 and 9, the authors could have suggested what further studies are necessary to address the suggested theories/findings.

General comments:

1. The paper has a few grammar and language issues, in the Abstract and Methods sections.

I have read this submission. I believe that I have an appropriate level of expertise to confirm that it is of an acceptable scientific standard.

# Response

**Published:** 2018-11-08

**Authors:** Endang Bachtiar

Dear editor and reviewer,

I am pleased to resubmit for publication the revised version of **Relationship between *Candida albicans *and *Streptococcus mutans* in early childhood caries, evaluated by quantitative PCR.**

We appreciate all the insightful comments provided by the reviewer. Based on the reviewer’s guidance, includes a number of positive changes, we endeavoured to improve the fit of the paper with the journal.

We hope that these revisions improve the paper such that the editor and reviewer now deem it worthy of publication in F1000 Research. Thank you for taking the time to help us improve the paper.

Sincerely,

Authors :  1. Endang W Bachtiar

                2. Boy M Bachtiar

Author’s response to reviewer comments

Comment provided by Reviewer #1

Methods:

Reviewer’s comment:

The authors did not mention how the examiners were calibrated, in order to reduce the inter- and intra-examiner variability when examining the preschool children, before grouping them into the early childhood caries and caries-free groups.

Author’s response:

We thank the reviewer for this correction. The problem has been fixed.

As suggested by the reviewer, we have added sentence within material and method as follow: “two weeks prior to collect to clinical samples, the examiners were calibrated and trained by providing with the manual describing study protocol and guidance regarding the examination of preschool children. Therefore, only those trained-examiners evaluated the preschool children”.

Reviewer’s comment:

The authors may have been able to collect more information on the oral hygiene habits or dietary intake of the subjects, before taking the oral samples.

Author’s response:

In this study, we used a questionnaire regarding the parent’s/ guardian’s perception concerning children’s general and oral health, the risk factor for caries, dietary intake, and access to dental care. We noticed, in general, most of the responders believed that dental decay is a natural phenomenon. However, considering the complexity of the issue studied in this study, it is not the authors’ intent to include oral hygiene habits or dietary intake of the subjects (preschool children). Therefore, the related-data were not included in the current study.

  

Reviewer’s comment:

In the caption for Figure 2, the symbol *p<0.05 should be indicated clearly. For Figure 2A, it is to show that there is a statistically significant difference between the *C. albicans* in saliva and dental plaque in the ECC group, and a statistically significant difference between the S. mutans in saliva and dental plaque in the ECC group. The use of top square bracket ( ⎴ ) with an asterisk would be better to indicate which parameters are statistically compared.

For Figure 4A, the use of top square bracket ( ⎴ ) with an asterisk would be better to indicate the parameters which are statistically compared.

Author’s response:

According to the suggestions provided by Reviewer#1, we have corrected the old figures. The top square bracket has been added in Fig. 2A to indicate a statistically significant difference as suggested.  For the Fig. 4A, we have added the top square bracket with an asterisk to indicate a significant difference between the parameters compared. Thank you.

Discussion:

Reviewer’s comment:

In paragraphs 3 and 7, the authors speculated that a high cariogenic/high sucrose concentration diet might influence the interaction between *C. albicans* and *S. mutans* in the tested subjects. The authors may have been able to draw a better suggestion if the information on the oral hygiene habits or diet is obtained during the recruitment of the subjects (See my comment in Method (2)).

Author’s response:

As mentioned above (response to the reviewer’s comment in methods (2); it is not the authors’ intent to include the oral hygiene habits or diet of the preschool children in this study. This is because the information regarding oral hygiene habit or diet, provided by the accompanying guardian are difficult to be understood.  Therefore, it is hard to interpret oral hygiene habit or diet data.

Reviewer’s comment:

In paragraphs 3, 7 and 9, the authors could have suggested what further studies are necessary to address the suggested theories/findings.

Author’s responses:

According to reviewer suggestion, we have added a better suggestion at the end of paragraph-3, as follow; “Further studies to obtain data regarding cariogenic diet are therefore necessary”. Additionally, we have added a sentence at the end of paragraph 7, as follow: “More studies regarding the involvement of gtfB expression, and how it relates to oral health conditions as well as cariogenic intake in Indonesian preschool children, are needed.

At the end of paragraph 9 (conclusion), as suggested by the reviewer, we added sentences; “Moreover, since the information regarding oral hygiene habit or diet, provided by the accompanying guardian are difficult to be understood, future studies involving preschool children may wish to involve examiners who are more experience in dealing with such parents/guardians when planning studies”. Thank you.

Dear Editor,

We are pleased to inform you that; 1/ for all the revision, as suggested by the reviewer, were showed in yellow-highlighting words, 2/ we revised the figure 2 and 4 to address the reviewer’s comments. Although the Fig. 3 is not included in reviewer’s comment, we revised the font, from the original (Arial) to new version (Times New Roman). Thus, all founds depicted in figures are similar. Thank you.
